# Differential mRNA Expression and Circular RNA-Based Competitive Endogenous RNA Networks in the Three Stages of Heart Failure in Transverse Aortic Constriction Mice

**DOI:** 10.3389/fphys.2022.777284

**Published:** 2022-03-07

**Authors:** Xiang Li, Weijiang Tan, Shuang Zheng, W. Glen Pyle, Caiyi Zhu, Honghua Chen, Le Kang, Jian Wu, Yunzeng Zou, Peter H. Backx, Feng Hua Yang

**Affiliations:** ^1^Guangdong Laboratory Animals Monitoring Institute, Guangdong Province Key Laboratory of Laboratory Animals, Guangzhou, China; ^2^College of Veterinary Medicine, South China Agricultural University, Guangzhou, China; ^3^Department of Biomedical Sciences, University of Guelph, Guelph, ON, Canada; ^4^Shanghai Institute of Cardiovascular Diseases, Zhongshan Hospital and Institutes of Biomedical Sciences, Fudan University, Shanghai, China; ^5^Department of Physiology, University of Toronto, Toronto, ON, Canada; ^6^Department of Biology, York University, Toronto, ON, Canada

**Keywords:** pressure overload, heart failure, mRNA, circRNA, ceRNA network

## Abstract

**Background:**

The murine transverse aortic constriction (TAC) model is frequently used to investigate molecular mechanisms underlying heart failure. However, limited data is available regarding the expression of mRNAs and circRNAs in murine heart failure progression induced by pressure overload.

**Methods:**

Transverse aortic constriction was used to induce pressure overload for 2, 4, and 8 weeks in mice. Echocardiographic measurements in B-mode and M-mode, as well as blood flow Doppler data were collected in mice without (sham) and with (2W-, 4W-, and 8W-post-TAC) pressure load. Hearts were excised and morphology, cardiomyocyte size, and fibrosis were determined. RNA sequencing, circRNA microarray, functional mRNA enrichment analysis, hub gene identification, target miRNA interaction, and competitive endogenous RNA (ceRNA) network construction were conducted.

**Results:**

Heart weight, cardiomyocyte hypertrophy, and fibrosis gradually increased over time in the hearts with pressure overload. The 2W-post-TAC hearts displayed concentric hypertrophy, thickened left ventricular walls, and increased EF and FS. The 4W-post-TAC hearts were characterized by preserved EF and FS, dilated atria, and increased left ventricle (LV) systolic volume. The 8W-post-TAC hearts presented with ventricular and atrial dilation, increased LV systolic and diastolic volume, reduced EF and FS, and increased ejection time (MV ET). mRNA expression analysis suggested that cardiac remodeling, immune response dysregulation, and metabolic disorder were the key cellular events in heart failure progression. Depression in chemotaxis and mitochondrial function were predicted in 4W- and 8W-post-TAC myocardia, respectively. A ceRNA network analysis demonstrated that the circRNAs targeted the expression of genes enriched in metabolism dysregulation in the 2W-post-TAC hypertrophic hearts, while they targeted genes enriched in cardiac remodeling in the 4W-post-TAC EF-preserved hearts and in the suppression of oxidative phosphorylation and cardiac contraction in the 8W-post-TAC EF-reduced hearts.

**Conclusion:**

Our work empirically demonstrates that distinctive features of heart failure, including ventricular hypertrophy, heart failure with preserved EF (HFpEF), and heart failure with reduced EF (HFrEF) are present in the murine pressure overload models. The three stages of heart failure vary in terms of mRNA and circRNA expression, as well as ceRNA regulation in a manner consistent with their structural, functional, and pathological differences.

## Introduction

Despite the improved preventative and therapeutic strategies, the prevalence of heart failure continues to increase worldwide. Hypertension represents a key risk factor of heart failure (Messerli et al., 2017). In fact, the Framingham Heart Study and Framingham Offspring Study revealed that 91% of patients with new-onset heart failure had a history of hypertension during the 20-year follow-up (Levy et al., 1996). Hypertension causes compensatory and decompensatory adaptation of the heart. The primary feature of the hypertension asymptomatic stage is compensatory hypertrophy of the left ventricle (LV) in response to elevated blood pressure. Echocardiographic evaluation has revealed that 19–48% of all untreated hypertensive patients present with left ventricular hypertrophy (Cuspidi et al., 2012). Subsequently, longstanding hypertension with sustained pressure overload culminates in left ventricular dilation and reduced ejection fraction (EF). Hypertensive hearts progress from left ventricular hypertrophy to heart failure [with preserved ejection fraction (HFpEF) or reduced ejection (HFrEF)] (Drazner, 2011). Guidelines provided by the American College of Cardiology/American Heart Association for the treatment of heart failure adopts a stage-based approach (Yancy et al., 2017). It is, therefore, imperative to distinguish between heart failure development stages, pathological characteristics, and molecular mechanisms in the treatment of heart failure in disease models.

Aortic constriction was reported as early as the 1950s to induce hypertension in rodents (Beznak, 1955). Among several constrict sites, transverse aortic constriction (TAC) is frequently used in animal studies to mimic pressure overload-induced cardiac remodeling and heart failure (Patten and Hall-Porter, 2009; Houser et al., 2012). Beznak stated that rats with aortic constriction rapidly develop ventricular hypertrophy. Distinct remodeling phenotypes in ventricular mass or systolic function may also be caused by chronic TAC induction, and/or limiting the extent of aortic constriction in mice (Mohammed et al., 2012; Richards et al., 2019). However, these inconsistencies in the phenotypes manifested among different research groups have restricted the use of this model. Hence, the murine TAC model must be precisely characterized to facilitate its application in the identification of fundamental causes of heart failure and the development of novel heart failure therapies.

The established mechanisms of pressure overload-induced heart failure include the dysregulation of hormonal peptides (Franco et al., 2004; Patel et al., 2005), signaling pathways (Dobaczewski et al., 2011), gene expression (Manabe et al., 2002), and metabolic processes (Gibb and Hill, 2018). Recently, non-coding circular RNAs (circRNAs) were reported to regulate heart failure progression (Li et al., 2018; Zhang et al., 2020). These non-coding RNA molecules are transcript isoforms that occur as covalently closed continuous loops that act as miRNA sponges, interact with RNA binding proteins, or are translated into proteins, exerting a broad range of effects on the heart (Altesha et al., 2019; Kristensen et al., 2019; Du et al., 2021). The most investigated function of circRNAs is miRNA sponging in which circRNAs and mRNAs competitively bind miRNAs to regulate gene expression, which is designated ceRNA regulation (Salmena et al., 2011; Hansen et al., 2013; Thomson and Dinger, 2016). An engineered circmiR acts as a sponge of miRNAs and attenuates pressure overload-induced cardiac hypertrophy (Lavenniah et al., 2020). However, limited research has been conducted on the roles of circRNA-based ceRNA networks in the progression of hypertensive heart failure.

In the present study, we explored the expression of mRNAs and circRNAs in the three stages of heart failure in TAC mice and identified distinct pathological features during heart failure progression. We found that HFpEF serves as the transition from hypertrophy to HFrEF. We analyzed mRNA and circRNA expression at three distinct heart failure stages and elucidated the circRNA-based regulation in heart failure. Our findings suggest that the ceRNA network can be an effective target for heart failure therapy.

## Materials and Methods

### Animals and Experimental Design

**FIGURE 1:**
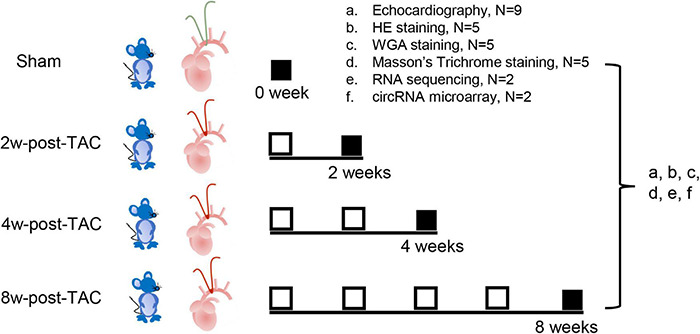
Experimental design. Thirty-six C57BL/6J mice underwent transverse aortic constriction (TAC) surgery. Following 2-, 4-, and 8 weeks TAC stress, heart structure and function at each stage were assessed by echocardiography (a; *N* = 9/group), histology examination (b, HE staining; c, WGA staining; d, Masson’s trichrome staining; *N* = 5/group), RNA sequencing (e; *N* = 2/group), and circRNA microarray analyses (f; *N* = 2/group).

Transverse aortic constriction surgery was performed on mice to induce pressure overload in their LVs. Thirty-six C57BL/6J male mice aged 3 months were randomly divided into four groups including the sham and the 2W-, 4W-, and 8W-post-TAC groups (*N* = 9). Heart structure and function at each stage were assessed by echocardiography, histological examination (*N* = 5/group), RNA sequencing, and circRNA microarray analyses (*N* = 2/group; Figure 1). The study was approved by the Institutional Animal Care and Use Committee (IACUC) of the Guangdong Laboratory Animals Monitoring Institute (No. IACUC2017015). The mice were maintained in a specific pathogen-free (SPF), AAALAC-accredited facility at the Guangdong Laboratory Animals Monitoring Institute [license No. SYXK(YUE)2016-0122; China]. The ambient temperature and humidity of the facility were 24 ± 2°C and 40–60%, respectively, with a 12 h day/12 h night light cycle.

### Transverse Aortic Constriction

Mice were anesthetized with 2% isoflurane. The animals were then subjected to tracheal intubation and connected to a ventilator to establish a gas path. Then 1.5% isoflurane was administered to maintain anesthesia. The surgical area was disinfected and a ∼1 cm incision was made around the second rib to open the thoracic cavity. Forceps were used to expose the aortic thoracic segment. To constrict the aorta, a 7-0 suture was installed under the aorta in the brachiocephalic trunk and the left common carotid artery, and a 26G blunt needle was inserted in parallel with the aorta. Surgical knots were tied, and the needle was removed. The sham group underwent the same surgical preparation but without aortic constriction.

### Echocardiography M-Mode, B-Mode, and Mitral Inflow Doppler

To evaluate changes in heart structure and function after TAC, a small animal ultrasound system with the MS-440 probe (Vevo 2100; VisualSonics, Toronto, ON, Canada) was applied. The mice in each group (sham, 2W-post-TAC, 4W-post-TAC, and 8W-post-TAC) were anesthetized with isoflurane and set in the supine position. Horizontal parasternal long axis B-mode images of the heart were obtained by placing the probe on the chest midline and pointing it toward the right shoulder. The probe was rotated clockwise by 90° to acquire images of the parasternal short-axis B-mode in the papillary muscle plane. The M-mode images were acquired at the same position as those of the B-mode recording. The structural indices included interventricular septum thickness at end-systole and diastole (IVS;s and IVS;d), left ventricular posterior wall thickness at end-systole and diastole (LVPW;s and LVPW;d), left ventricular internal dimension at end-systole and diastole (LVID;s and LVID;d), atrial area at long-axis cross-section, and aortic root diameter. The function indices included cardiac output (CO), ejection fraction (EF), and short axis fractional shortening (FS).

A mitral inflow Doppler tool was used to evaluate cardiac diastolic function. The probe was placed on the lateral thoracic wall to obtain a four-chamber view of the heart apex. The Doppler sampling volume cursor was then moved to the mitral valve cusp and the early diastolic blood flow peak E-wave velocity (MV E), the late diastolic blood flow peak A-wave velocity (MV A), isovolumetric contraction time (IVCT), isovolumetric relaxation time (IVRT), and ejection time (MV ET) were measured.

### Hematoxylin and Eosin, Wheat Germ Agglutinin, and Masson’s Trichrome Staining

Pathological examinations were conducted to evaluate tissue damage, cardiomyocyte size, and cardiac fibrosis as previously described (Li X. et al., 2020; Tan et al., 2021). Hematoxylin and Eosin (HE) staining detects the nuclei, cardiomyocytes, and morphology of the heart. Wheat Germ Agglutinin (WGA) conjugated with fluorescein labels the glycoproteins to visualize cardiomyocyte plasma membranes in fixed heart tissue. Masson’s trichrome staining selectively reveals collagen fibers (blue), muscle tissue (red), and nuclei (black). Mice from all four groups were euthanized and their hearts were excised, fixed for 12–16 h in 4% (v/v) paraformaldehyde (PFA), embedded in paraffin, and longitudinally sectioned into 3 μm slices.

Hematoxylin and eosin staining was performed as follows: (1) sections were dewaxed with xylene, (2) dehydrated with an ethanol concentration gradient series, (3) stained with hematoxylin solution (No. H3136; Sigma-Aldrich Corp., St. Louis, MO, United States), (4) differentiated with HCl in ethanol, (5) stained with alcohol-soluble eosin (No. E4009; Millipore Sigma Corp., Burlington, MA, United States), and (6) dehydrated and mounted. For the WGA staining, fluorescein-labeled WGA (No. W11261; Invitrogen, Carlsbad, CA, United States) was added to the tissue sections, which were incubated at room temperature (20–25°C) in the dark for 10 min. After staining, the sections were washed twice with phosphate-buffered saline (PBS) and mounted with 4′,6-diamidino-2-phenylindole (DAPI; No. P36962; Invitrogen).

For Masson’s trichrome staining, the dewaxed sections were fixed in Bouin’s solution (No. HT10132; Millipore Sigma Corp., Burlington, MA, United States) overnight and stained with Weigert’s hematoxylin (No. HT10132; Millipore Sigma Corp., Burlington, MA, United States) and Biebrich scarlet-acid fuchsin solution (No. HT151; Millipore Sigma Corp., Burlington, MA, United States) for 3–5 min. Then 1% (w/v) phosphomolybdic acid was applied to the sections to wash out excess stain; sections were then incubated in Aniline blue (No. B8653; Millipore Sigma Corp., Burlington, MA, United States) for ∼5 min, dehydrated, and mounted. Pathological changes in pressure overload hearts were identified microscopically (DM2500; Leica Microsystems, Wetzlar, Germany). The cardiomyocytes were measured with ImageJ 1.52a (NIH, Bethesda, MD, United States).

### mRNA Sequencing, Raw Data Processing, and Differentially Expressed Genes

mRNA sequencing was used to profile gene expression at the hypertrophic, HFpEF, and HFrEF stages in murine pressure overload hearts, as previously described (Li X. et al., 2020; Tan et al., 2021). Total RNA was isolated with TRIzol reagent (Thermo Fisher Scientific, Waltham, MA, United States). The RNA concentrations were determined with a Qubit 2.0 fluorometer and a Qubit assay kit (Life Technologies, Waltham, MA, United States). RNA integrity was assessed with the RNA Nano 6000 assay kit and a Bioanalyzer 2100 system (Agilent Technologies, Santa Clara, CA, United States). Sequencing libraries were generated with the NEBNext Ultra RNA library prep kit for Illumina (New England Biolabs, Ipswich, MA, United States). After index codes were added, the samples were clustered with a cBot cluster generation system and a TruSeq PE cluster kit v3-cBot-HS (Illumina, San Diego, CA, United States). An Illumina HiSeq platform (Illumina) was used to sequence the libraries and generated 125 bp/150 bp paired-end reads. Raw data with paired-end clean reads were mapped for further analysis. The mRNA-Seq data were uploaded to the gene expression omnibus (GEO) database (accession No. GSE182985).^1^

The original reads of the genes in the samples from the 2W-post-TAC, 4W-post-TAC, and 8W-post-TAC were compared against those of the sham group. Fold changes between paired groups were log2-transformed. *P* < 0.05 was applied as the cutoff for differentially expressed gene (DEG) identification. DEGs with log2 (fold change) > 1 were considered upregulated while those with log2 (fold change) < −1 were downregulated. Six DEG datasets were defined: one upregulated and one downregulated dataset each for 2W-post-TAC vs. sham, 4W-post-TAC vs. sham, and 8W-post-TAC vs. sham.

### Gene Set Enrichment Analysis

Gene Set Enrichment Analysis (GSEA) was used to predict the potential roles of DEGs in LV hypertrophy, HFpEF, and HFrEF of TAC mice. The six sets of upregulated and downregulated DEGs were uploaded to the web-accessible gene annotation and the Metascape analytical tool.^2^ A significant BP term included ≥3 candidates, *P* ≤ 0.05, ≥1.5 enrichment factor, or a limiting-network interactome consisting of 3–500 candidate proteins (Zhou et al., 2019). The high *P*-value (−log10) of the BP term reflected its relatively higher degree of gene enrichment. Each DEG in the interaction network was displayed in the modules according to its biological processes.

### Real-Time Quantitative PCR

Three DCRs in each heart failure stage were subjected to Quantitative PCR (qPCR) to verify their expression. The protocol was carried out as previously described (Li X. et al., 2020). The primers used in this study are shown in Supplementary Table 1. TB Green Premix Ex Taq II (No. RR820; Takara Bio Inc., Japan) was used. The program was as follows: 95°C for 30 s (1 cycle); 95°C for 5 s, 60°C for 34 s (40 cycles); and 72°C for 10 min (1 cycle). Gene expression levels were normalized to β-actin.

### Hub Gene Identification

A protein-protein interaction (PPI) network analysis was performed to identify hub genes. This analysis consisted of two steps and elucidated the altered expression and roles of genes in structural and functional remodeling in 2W, 4W, and 8W pressure overload myocardia. Total DEGs for each comparison pair (2W-post-TAC vs. sham, 4W-post-TAC vs. sham, or 8W-post-TAC vs. sham) were uploaded to the Search Tool for the Retrieval of Interacting Genes/Proteins database (STRING 11.0)^3^ to construct a protein-protein interaction (PPI) network. In this manner, the types and intensities of the interactions between proteins encoded by the DEGs were established. The CytoHubba plug-in of Cytoscape^4^ including the maximal clique centrality (MCC) scoring method was used to capture the essential nodes in the PPI network (Chin et al., 2014). The top 40 nodes ranked by the MCC algorithm were considered hub genes and subsequently used to predict miRNAs interacting with mRNAs. Diagrams of the PPI network and the hub gene sub-network were edited and viewed in Cytoscape v. 3.8.1 (see text footnote 4).

### Prediction of mRNA–miRNA Interaction

The miRNAs targeting the mRNAs were then predicted. In the present study, a newly updated miRWalk database with the Python Django framework was used to predict miRNAs. This framework incorporates datasets from TargetScan (v. 7.1; conserved site context scores), miRDB (release 5.0), with validated information from miRTarBase v. 7.0 (Sticht et al., 2018). For data analysis, the top 40 hub genes from each comparison pair (2W-post-TAC vs. sham, 4W-post-TAC vs. sham, and 8W-post-TAC vs. sham) were uploaded into the miRWalk database. As each mRNA can be targeted by multiple miRNAs, the criteria for selecting each miRNA were *P* ≥ 95% for the 3′-UTR miRNA-binding sites, and at least one occurrence of the miRNA in TargetScan, miRDB, or miRTarBase.

### Circular RNA Microarray, Chip Data Preprocessing, and Differentially Expressed Circular RNAs

Arraystar mouse circular RNA array V2 (Agilent Technologies, Santa Clara, CA, United States) was used to profile circRNA expression in murine hearts during remodeling and dysfunction progression. After isolation, the total RNAs were digested with RNase R (Epicentre Technologies Corp., Madison, WI, United States) to remove linear RNAs and enrich circular RNAs. The latter were further amplified and transcribed by random priming into fluorescent cRNA (Arraystar Super RNA labeling kit; Agilent Technologies, Santa Clara, CA, United States). The labeled cRNA concentration and activity in pmol Cy3/μg cRNA were measured. One microgram of each labeled cRNA was fragmented, heated, and assembled onto the Arraystar Mouse circular RNA Array V2 (8 × 15K; Agilent Technologies, Santa Clara, CA, United States). The arrays were hybridized, washed, fixed, scanned with an Agilent scanner (No. G2505C; Agilent Technologies, Santa Clara, CA, United States), and analyzed with Agilent feature extraction software v. 11.0.1.1 (Agilent Technologies, Santa Clara, CA, United States). Microarray data were uploaded to the gene expression omnibus (GEO) database (see text footnote 1) under accession No. GSE182912. The array images were imported into the database followed by quantile intensity normalization of chips and samples in an Arraystar’s analysis package of R (R Core Team, Vienna, Austria).

The datasets of the 2W-, 4W-, and 8W-post-TAC arrays were compared against that of the sham array in the circRNA microarray data analyses. Fold changes in circRNA expression of each pair (2W-post-TAC vs. sham, 4W-post-TAC vs. sham, and 8W-post-TAC vs. sham) were then determined. Differentially expressed circRNAs were designated “DECRs”. *P* < 0.05 was the cutoff for DECR definition. DEGs with log2 (fold change) > 1 were considered upregulated while those with log2 (fold change) < −1 were considered downregulated. The top 40 DECRs (20 upregulated and 20 downregulated) associated with each heart failure stage (2W-, 4W-, and 8W-post-TAC) were selected to predict circRNA-miRNA interactions.

### Prediction of Circular RNA–miRNA Interactions

The Arraystar miRNA database with the datasets from TargetScan [4] and miRanda [5] was used to predict the circRNA-miRNA interactions. TargetScan can be searched for conserved 8mer, 7mer, and 6mer sites matching the seed region of ≥1 miRNA (Lewis et al., 2005) as well as sites with mismatches in the seed region. The latter are compensated by conserved 3′ pairing and centered sites (Friedman et al., 2009; Shin et al., 2010). The predicted targeting efficacy is evaluated and ranked by site score (Agarwal et al., 2015) and probability of conserved targeting (PCT) (Friedman et al., 2009). The miRanda is based on sequencing features, thermostability, and evolutionary conservation and predicts miRNA targets (Betel et al., 2008). Here, total DECRs (up- and downregulated) for each comparison pair (2W-post-TAC vs. sham, 4W-post-TAC vs. sham, or 8W-post-TAC vs. sham) were uploaded to the Arraystar miRNA database, and the top five miRNAs sponged by each circRNA target were selected. Certain miRNAs were targeted by multiple circRNAs, and duplicates were discarded. Potential miRNAs targeted by each of the top 20 upregulated and the top 20 downregulated circRNAs were selected for subsequent analysis.

### Construction of Competitive Endogenous RNA Regulatory Networks

To understand the potential regulatory mechanisms of circRNAs after predicting mRNA–miRNA and circRNA–miRNA pairs, miRNAs common to both interaction pairs were selected, and the networks were combined and displayed. Below are the steps involved in constructing the circRNA–miRNA–mRNA network.

First, the miRNA groups were defined. (1) Based on the predictions of the mRNA–miRNA interactions, the miRNAs degrading the upregulated or downregulated mRNAs were designated “mRNA up” and “mRNA down”, respectively. That is, the six miRNA groups were “mRNA up” or “mRNA down” for 2W-post-TAC, 4W-post-TAC, and 8W-post-TAC. (2) Based on predictions of the circRNA-mRNA interactions, miRNAs sponged by upregulated or downregulated circRNAs were designated “circRNA up” and “circRNA down”, respectively. Thus, the six miRNA groups were “circRNA up” and “circRNA down” for 2W-post-TAC, 4W-post-TAC, and 8W-post-TAC.

The predicted miRNAs (“mRNA up,” “mRNA down,” “circRNA up,” or “circRNA down”) associated with each heart failure stages (2W-post-TAC, 4W-post-TAC, and 8W-post-TAC) were uploaded to the TBtools program (Chen et al., 2020) and subjected to Venn diagram analysis, generating one diagram per heart failure stage. In each Venn diagram, the intersection between the miRNA “mRNA up” and the circRNA “circRNA up” groups was considered to be associated with the circRNA-based upregulation of mRNAs. In contrast, in the same Venn diagram, the intersection between the miRNA “mRNA down” and the cicRNA “circRNA down” was considered the mRNAs involved in the circRNA-based downregulation of mRNAs. Thus, we determined the circRNAs that potentially up- or downregulated mRNA expression in the three stages of heart failure. The ceRNA network and the interactions among the circRNA-miRNA-mRNA were visualized in Cytoscape v. 3.8.1 (see text footnote 4).

### Statistical Analysis

For the echocardiographic and pathological examinations, the data are presented as means ± SEM. Raw data for the sham and the 2W-post-TAC, 4W-post-TAC, and 8W-post-TAC groups were uploaded to GraphPad Prism 8.0 (GraphPad Software, La Jolla, CA, United States). Significant differences among multiple data groups were analyzed by one-way ANOVA followed by Tukey’s multiple comparisons test (GraphPad Prism 8.0; GraphPad Software). Graphs showing each individual data point were plotted. For the mRNA sequencing and circRNA microarray, *t*-tests were used to identify significant differences between pairs of means. *P* < 0.05 was considered statistically significant.

## Results

### Heart Failure Through the Hypertrophy, Heart Failure With Preserved Ejection Fraction, and Heart Failure With Reduced Ejection Fraction Stages in Pressure Overload Hearts

**FIGURE 2:**
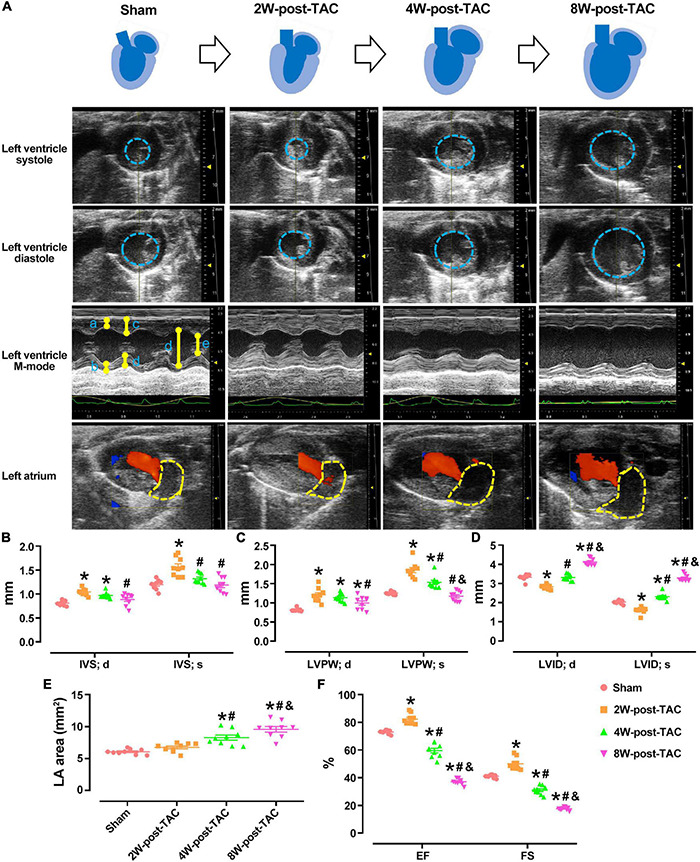
Echocardiography of the sham and 2W-, 4W-, and 8W-post-TAC animals. The structural changes in the sham and 2W-, 4W-, and 8W-post-TAC animals were displayed in panel **(A)**. The heart became hypertrophic within the first 2 weeks after surgery. The thicknesses of the internal septum and posterior walls had significantly increased. After the pressure overload had continued for 4 weeks, the heart became enlarged, as indicated in the area enclosed by the blue dashed lines. The left ventricles remained enlarged and by 8W post-surgery **(B,C)**. The internal diameters in the 2W-post-TAC group had significantly decreased, while they had increased in the 4W-post-TAC group compared with those of the 2W-post-TAC group. The internal systolic diameters in the 8W-post-TAC group were ∼1.5-fold greater than those of the sham ventricles **(D)**. Enlargement of the left atria, as indicated in the area enclosed by the yellow dashed lines, were observed in the 4W- and 8W-post-TAC group **(E)**. The functional EF indices had significantly increased, preserved, and decreased in the 2W-, 4W-, and 8W-post-TAC groups, respectively **(F)**. All data are presented as the means ± SEM. *N* = 9. *P < 0.05 vs. Sham, ^#^*P* < 0.05 vs. 2W-post-TAC, and ^&^*P* < 0.05 vs. 4W-post-TAC.

In this study, TAC surgery was used to induce left ventricular pressure overload, while heart structure and function were monitored at 2, 4-, and 8-weeks post TAC surgery. Echocardiography revealed that the peak flow velocity at the ascending aorta and diameter of the aortic root were increased significantly after TAC surgery (Table 1). The heart became hypertrophic within the first 2 weeks after surgery. The thicknesses of the internal septum and posterior walls had significantly increased while their internal diameters decreased (Figures 2A–D). After the pressure overload had continued for 4 weeks, the heart became enlarged. The thicknesses of the ventricular walls had decreased, and their internal diameters increased compared with those of the 2W-post-TAC group (Figures 2A–D). Left atrial enlargement was observed in the 4W-post-TAC group (Figures 2A,E). The LVs remained enlarged 8W post-surgery with the internal systolic diameters of the TAC ventricles ∼1.5-fold greater than those of the sham ventricles. Moreover, the size of the left atria had also significantly increased while the thicknesses of the ventricular walls were not changed (Figures 2A–E). The ventricular volumes at systole were gradually increased during heart failure progression, while at end-diastole the ventricular volumes were reduced, relatively normal, and elevated in the 2W-, 4W-, and 8W-post-TAC groups, respectively. Cardiac output was significantly decreased in the 4W- and 8W-post-TAC groups (Table 1). Furthermore, the functional EF indices had significantly increased (79–88%), were stable (55–66%), or decreased (33–40%) in the 2W-, 4W-, and 8W-post-TAC groups, respectively (Figure 2F and Table 1), suggesting the cardiac function progressed from compensatory to decompensatory. Hence, EF-preserved cardiac dysfunction is the transition from hypertrophy to HFrEF in murine pressure overload hearts. All measurements are listed in Table 1.

**TABLE 1:** Echocardiographic measurements.

Time	Sham	2W-post-TAC	4W-post-TAC	8W-post-TAC
Heart rate (beats/min)	443 ± 5	460 ± 9	435 ± 8	460 ± 7
Peak flow velocity at ascending aorta (mm/s)	−697.56 ± 22.68	−4519.87 ± 120.54*	−5087.78 ± 211.12*	−4806.48 ± 211.07*
Diameter of aortic root (mm)	1.39 ± 0.02	1.77 ± 0.04*	1.63 ± 0.02*^#^	1.80 ± 0.03*^&^
Interventricular ventricular septum thickness, end-systolic, IVS; s (mm)	1.20 ± 0.03	1.57 ± 0.06*	1.32 ± 0.04^#^	1.19 ± 0.05^#^
Interventricular ventricular septum thickness, end-diastolic, IVS; d (mm)	0.81 ± 0.02	1.04 ± 0.02*	0.97 ± 0.02*	0.89 ± 0.04^#^
Left ventricular posterior wall thickness, end-systolic, LVPW; s (mm)	1.25 ± 0.01	1.85 ± 0.07*	1.53 ± 0.06*^#^	1.17 ± 0.04^#&^
Left ventricular posterior wall thickness, end-diastolic, LVPW; d (mm)	0.81 ± 0.01	1.21 ± 0.06*	1.14 ± 0.04*	1.00 ± 0.06*^#^
Left ventricular internal dimension, end-systolic, LVID; s (mm)	2.03 ± 0.03	1.60 ± 0.05*	2.32 ± 0.06*^#^	3.29 ± 0.06*^#&^
Left ventricular internal dimension, end-diastolic, LVID; d (mm)	3.32 ± 0.05	2.83 ± 0.04*	3.31 ± 0.06^#^	4.12 ± 0.06*^#&^
Ventricular volume at systole (μL)	12.23 ± 0.31	6.39 ± 0.48*	17.49 ± 1.08*^#^	42.68 ± 1.68*^#&^
Ventricular volume at diastole (μL)	44.61 ± 1.50	35.23 ± 1.04*	43.14 ± 1.41	68.36 ± 2.59*^#&^
Cardiac output (ml/min)	14.47 ± 0.48	13.93 ± 0.64	10.31 ± 0.20*^#^	12.38 ± 2.55*^&^
LV Mass (corrected), mg	69.97 ± 2.09	93.90 ± 5.26*	104.03 ± 3.62*	124.86 ± 5.73*^#&^
Ejection fraction, EF (%)	73.03 ± 0.43	82.27 ± 1.27*	59.57 ± 1.64*^#^	37.10 ± 0.70*^#&^
Short axis fractional shortening, FS (%)	41.04 ± 0.37	49.98 ± 1.47*	31.02 ± 1.04*^#^	17.61 ± 0.38*^#&^
Area of left atria, LA (mm^2^)	6.06 ± 0.14	6.73 ± 0.22	8.27 ± 0.40*^#^	9.58 ± 0.43*^#&^
Peak E-wave velocity, MV E (mm/s)	621.09 ± 13.47	456.05 ± 14.86*	496.09 ± 24.52*	644.08 ± 14.76^#&^
Peak A-wave velocity, MV A (mm/s)	390.09 ± 16.92	430.16 ± 24.16	437.21 ± 17.59	354.88 ± 21.11^&^
Isovolumetric contraction time, IVCT (ms)	15.11 ± 0.80	10.33 ± 0.74*	11.10 ± 0.80*	14.44 ± 1.25^#^
Isovolumetric relaxation time, IVRT (ms)	21.60 ± 0.73	15.87 ± 0.62*	18.59 ± 0.94	21.34 ± 1.61^#^
Ejection time, MV ET (ms)	54.80 ± 0.89	44.43 ± 0.75*	51.80 ± 1.25^#^	62.29 ± 1.57*^#&^
Ratio of MV E to A	1.61 ± 0.06	1.08 ± 0.07*	1.14 ± 0.06*	1.85 ± 0.08^#&^

N = 8–9. All data are presented as the means ± SEM. *P < 0.05 vs. Sham, ^#^P < 0.05 vs. 2W-post-TAC, and ^&^P < 0.05 vs. 4W-post-TAC (one-way ANOVA, Tukey’s multiple comparisons test).

**FIGURE 3:**
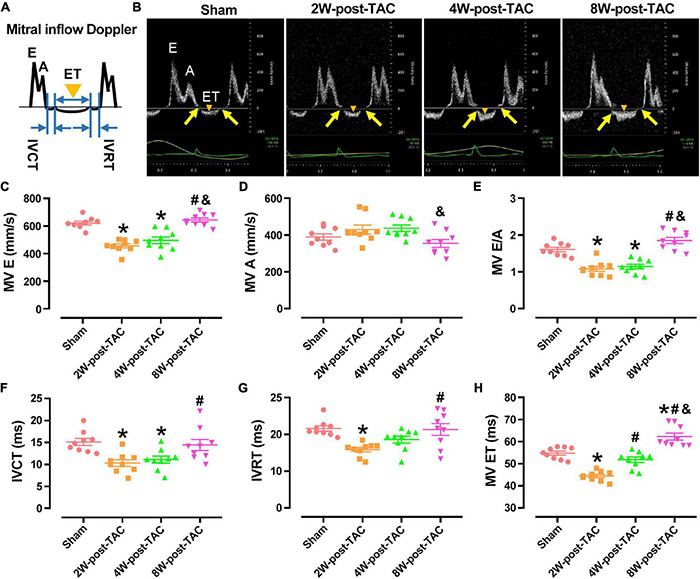
Indices of mitral inflow Doppler. A carton of and representing images of mitral inflow Doppler was displayed in panels **(A,B)**, respectively. The peak E-wave velocity (MV E) had decreased in the 2W and 4W-post-TAC groups **(C)**. The peak A-wave velocity (MV A) did not significantly differ among groups **(D)**. The MV E: A ratios had decreased in the 2W- and 4W-post-TAC groups but non-significantly increased (*P* = 0.0786) in the 8W-post-TAC group **(E)**. The isovolumetric contraction time (IVCT; **F**), isovolumetric relaxation time (IVRT; **G**), and ejection times (MV ET; **H**) had decreased in the 2W-post-TAC groups but elevated in the 8W post-surgery groups. The IVCT had also decreased in the 4W-post-TAC group **(F)**. Arrows in yellow, IVCT or IVRT; triangles in orange, MV ET; All data are presented as the means ± SEM. *N* = 8–9. *P < 0.05 vs. Sham, ^#^*P* < 0.05 vs. 2W-post-TAC, and ^&^*P* < 0.05 vs. 4W-post-TAC.

A mitral inflow Doppler showed that the MV E decreased in the 2W and 4W-post-TAC groups, while increasing in the 8W post-TAC group. The MV A did not significantly differ among groups (Figures 3A–D). The E:A ratios had decreased in the 2W- and 4W-post-TAC groups, while non-significantly increasing (*P* = 0.0786) in the 8W-post-TAC group (Figures 3B,E). The IVCT, IVRT, and MV ET decreased in the 2W-post-TAC groups and increased in the 8W post-surgery groups (Figures 3F–H). The IVCT also decreased in the 4W-post-TAC group (Figure 3F). All measurements are listed in Table 1.

### Pathological Characteristics of the Heart During Hypertrophy, Heart Failure With Preserved Ejection Fraction, and Heart Failure With Reduced Ejection Fraction

**FIGURE 4:**
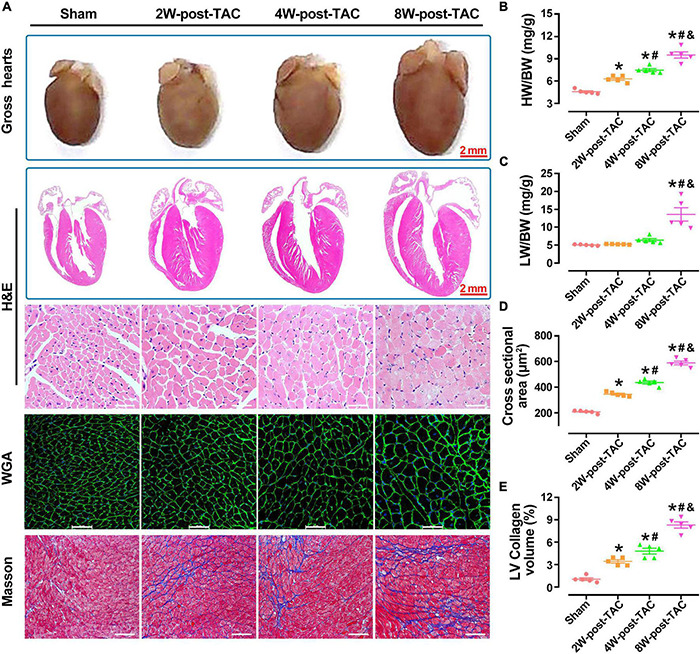
Pathological changes in the three stages of heart failure. A pathological examination showed that the morphological changes in heart size were consistent with the B-mode echocardiographic measurements of the sham, 2W-, 4W-, and 8W-post-TAC groups **(A)**. In the 2W-post-TAC group, the hearts presented with concentric hypertrophy **(A)** and the heart weight:body weight ratios had gradually increased **(B)**. The lung weight:body ratios increased in the 8W-post-TAC group **(C)**. High magnification of the HE staining revealed that the cardiomyocytes were hypertrophic in the 2W-, 4W-, and 8W-post-TAC groups **(A)**. The WGA staining of the cardiomyocyte membranes confirmed that pressure overload-induced cardiomyocyte hypertrophy and cardiomyocyte size gradually increased with pressure overload **(A,D)**. Fibrosis in the left ventricles gradually increased as hearts progressed from the hypertrophic to the HFpEF to the HFrEF stages **(E)**. All data are presented as the means ± SEM. *N* = 5. *P < 0.05 vs. Sham, ^#^*P* < 0.05 vs. 2W-post-TAC, and ^&^*P* < 0.05 vs. 4W-post-TAC.

A pathological examination showed that the changes in heart size were consistent with the B-mode echocardiographic measurements of the sham, 2W-, 4W-, and 8W-post-TAC groups (Figure 4A). In the 2W-post-TAC group, the hearts presented with concentric hypertrophy and the heart weight:body weight ratios gradually increased (Figure 4B). The lung weight:body weight ratios increased in the 8W-post-TAC group (Figure 4C), indicating that lung dysfunction might present in pressure overload-induced HFrEF in mice. Longitudinal HE-stained heart sections further revealed that with extended pressure overload, the internal dimensions of the left atria and ventricles expanded in the 4W- and 8W-post-TAC hearts compared with the sham and 2W-post-TAC hearts. The morphological changes observed in the anatomical and histological examination were consistent with that measured by echocardiography. Furthermore, high magnification of the HE staining revealed that the cardiomyocytes were hypertrophic in the 2W-, 4W-, and 8W-post-TAC groups. The WGA staining of the cardiomyocyte membranes confirmed that pressure overload-induced cardiomyocyte hypertrophy and cardiomyocyte size gradually increased with pressure overload (Figures 4A,D). Fibrosis in the LVs gradually increased as hearts progressed from the hypertrophic to the HFpEF and subsequent HFrEF stages (Figures 4A,E).

### Differential mRNA and Circular RNA Expression Profiles in Post-transverse Aortic Constriction Mouse Hearts

**FIGURE 5:**
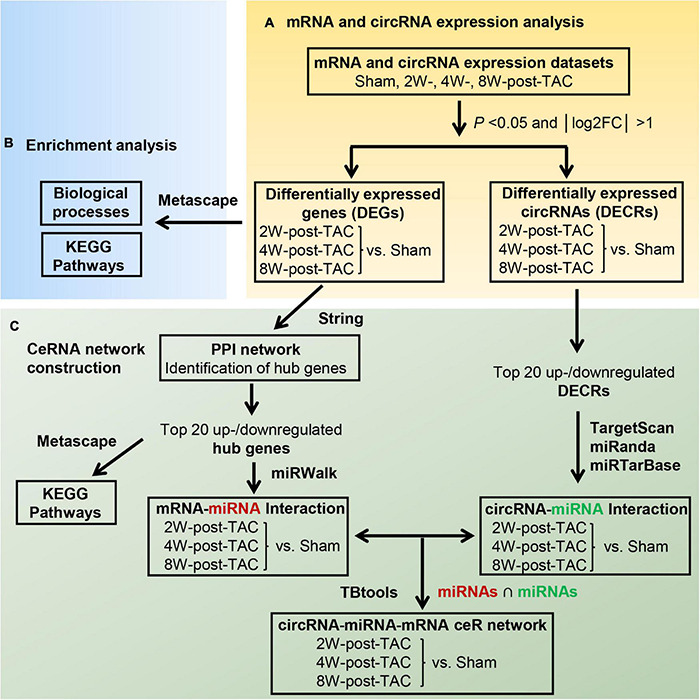
Flow diagram of the bioinformatics analyses. The analyses of the differential expression profiles of mRNAs and circRNAs in murine 2W-, 4W-, and 8W-post TAC hearts was shown in panel **(A)**. After characterizing the mRNA expression profiles, the DEGs were subjected to functional enrichment analysis using Metascape **(B)**. The ceRNA network construction procedure for 2W-, 4W-, and 8W-post TAC hearts **(C)** included (1) the top 40 hub genes were identified by PPI network analysis in the three stages of heart failure and hub gene enrichment analysis by Metascape, (2) Top 20 upregulated and 20 downregulated hub genes were proceeded to predict the miRNAs that inhibit the mRNAs of these hub genes. (3) Top 20 upregulated and 20 downregulated DECRs were subjected to predict the miRNAs that bind to circRNAs. (4) The intersection of miRNAs predicted from two interactions of “mRNA-miRNAs” and “circRNA-miRNA” served as an intermediator for the circRNA-based ceRNA regulation on mRNAs.

**FIGURE 6:**
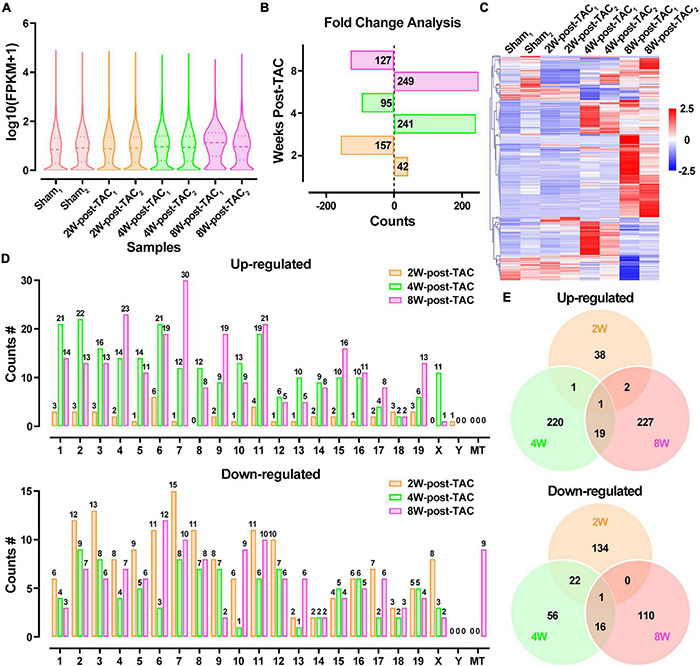
Differential expression profiles of mRNAs in the murine post-TAC hearts. **(A)** The distributions of the total mRNAs in each sample. **(B)** Counts of up- and downregulated differential expressed genes (DEGs) in the 2W-, 4W-, and 8W-post-TAC groups. **(C)** Clustering heatmap plot of DEGs showed 24 main clusters for eight heart samples (two of each heart failure groups). Upregulated (red) or downregulated (blue) DEGs were distinguished from different heart failure stages. **(D)** The numbers of differentially expressed circRNAs (DECRs) distributed in murine chromosomes and mitochondria. **(E)** Venn diagrams illustrated the distinction and overlapping of the up- and downregulated differential expressed circRNAs in the in the 2W-, 4W-, and 8W-post-TAC groups. The cutoff for the DEGs was *P* < 0.05 and | log2FC | > 1. *N* = 2 for the sham, 2W-, 4W-, and 8W-post-TAC groups.

The transcript expression analyses in murine TAC hearts followed the procedure shown in Figure 5A. Distributions of total DEG expression in the 2-, 4-, and 8-week post-TAC myocardia are displayed in Figure 6A. A total of 199 (42 upregulated and 157 downregulated), 336 (241 upregulated and 95 downregulated), and 376 (249 upregulated and 127 downregulated) DEGs were detected in the hearts 2-, 4-, and 8-weeks post-TAC, respectively, compared with the sham hearts (Figure 6B). A clustered heatmap analysis showed that the DEG expression patterns were different between the four experimental groups (Figure 6C). The number of DEGs in the same chromosome regions differed among the hypertrophy, HFpEF, and HFrEF stages (Figure 6D). Venn diagrams of the upregulated and downregulated DEGs showed that only two (one upregulated, one downregulated) coexisted in the intersections of the 2W-post-TAC vs. 4W-post-TAC vs. 8W-post-TAC groups (Figure 6E).

**FIGURE 7:**
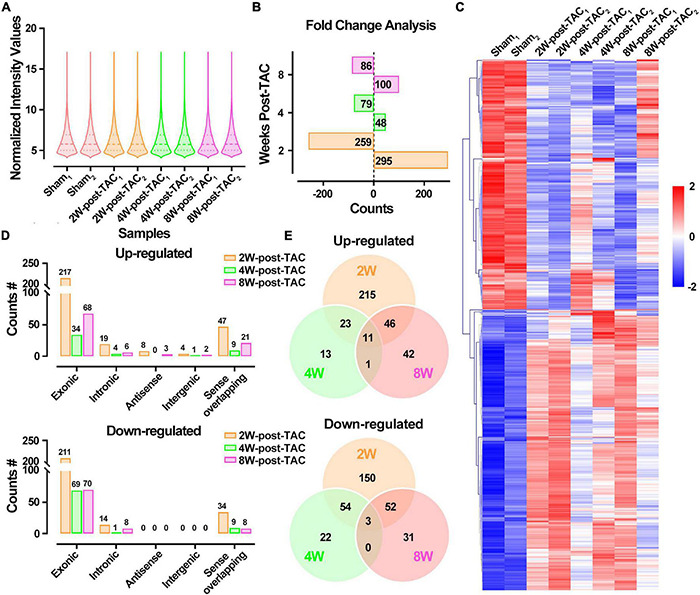
Differential expression profiles of circRNAs in the murine post-TAC hearts. **(A)** The distributions of the total circRNAs in each sample. **(B)** Counts of up and downregulated differential expressed circRNAs (DECRs) in the 2W-, 4W-, and 8W-post-TAC groups. **(C)** Clustering heatmap plot of DEGRs showed 12 main clusters for eight heart samples (two of each heart failure groups). Upregulated (red) or downregulated (blue) DECRs were distinguished from different heart failure stages. **(D)** The numbers of differentially expressed circRNAs (DECRs) in exonic, intronic, antisense, intergenic, and sense overlapping circRNAs. The colors of yellow, green, and pink represent 2W-, 4W-, and 8W-post-TAC groups, respectively. **(E)** Venn diagrams illustrated the distinction and overlapping of the up- and downregulated differential expressed circRNAs in the 2W-, 4W-, and 8W-post-TAC groups. The cutoff for DECRs was *P* < 0.05 and | log2FC | > 1. *N* = 2 for the sham, 2W-, 4W-, and 8W-post-TAC groups.

The DECR expression patterns of the 2W-, 4W-, and 8W-post TAC myocardia are illustrated in Figure 7A. There were 295 upregulated and 259 downregulated DECRs in the 2W-post-TAC hypertrophic group relative to the sham group. In the 4W-post-TAC HFpEF group, 48 upregulated and 79 downregulated DECRs were identified compared with the sham group. In the 8W-post-TAC HFrEF group, 100 upregulated and 86 downregulated DECRs were detected relative to the sham group (Figure 7B). A clustered heatmap showed that the circRNA expression patterns differed between the sham and TAC groups (Figure 7C). The numbers of DECRs within the same chromosome regions also differed between the sham and TAC groups (Figure 7D). A Venn diagram analysis revealed 11 upregulated and three downregulated DECRs in the interactions between 2W-post-TAC DECRs vs. 4W-post-TAC DECRs vs. 8W-post-TAC DECRs (Figure 7E).

### Heart Failure Is Regulated in Both the Same and Different Ways at Each Stage

**FIGURE 8:**
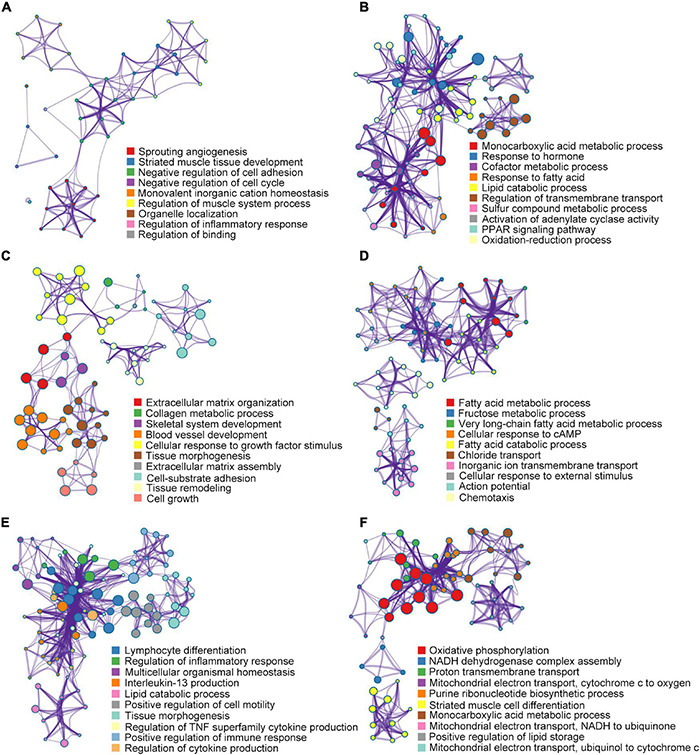
Gene set enrichment analysis for DEGs. In the 2W-post-TAC hypertrophic hearts, a biological process enrichment analysis of the DEGs (2W-post-TAC vs. sham) revealed augmentation mainly in cardiac remodeling and immune response **(A)**, and suppression in metabolism and cardiac regulation **(B)**. In the 4W-post-TAC HFpEF groups, the DEGs (4W-post-TAC vs. sham) were found to be augmented in cardiac remodeling, and suppressed in metabolism **(C)**, contractile regulation and immune response **(D)**. In the 8W-post-TAC HFrEF hearts, the enhanced biological processes included immune response, cardiac remodeling, and lipid catabolism **(E)**, and the depressed biological processes included mitochondrial function, cardiomyocyte growth and lipid storage **(F)**. *N* = 2 for the sham, 2W-, 4W-, and 8W-post-TAC groups. *N* = 2 for the sham, 2W-, 4W-, and 8W-post-TAC groups.

After characterizing the mRNA expression profiles, the DEGs were subjected to enrichment analysis using Metascape (Figure 5B and Supplementary Table 2). During the hypertrophic stage, the GSEA of the DEGs (2W-post-TAC vs. sham) revealed that the augmented biological processes included cardiac remodeling (sprouting angiogenesis, striated muscle tissue development, negative regulation of cell cycle, regulation of muscle system process, organelle localization, and regulation of binding) and immune response (negative regulation of cell adhesion, regulation of inflammatory response; Figure 8A). The suppressed DEGs were enriched in metabolism (monocarboxylic acid metabolic process, response to hormone, cofactor metabolic process, response to fatty acid, lipid catabolic process, sulfur compound metabolic process, PPAR signaling pathway, and oxidation-reduction process) and cardiac contractile regulation (regulation of transmembrane transport, activation of adenylate cyclase activity; Figure 8B).

During the HFpEF stage of pressure overload hearts, the DEGs (4W-post-TAC vs. sham) were found to be enriched in cardiac remodeling, metabolism, and functional regulators (Figures 8C,D). Augmented biological processes were associated with cardiac remodeling (extracellular matrix organization, collagen metabolic process, skeletal system development, blood vessel development, cellular response to growth factor stimulus, tissue morphogenesis, extracellular matrix assembly, cell-substrate adhesion, tissue remodeling, and cell growth). The suppressed biological processes were associated with metabolism (fatty acid metabolic process, fructose metabolic process, very long-chain fatty acid metabolic process, and fatty acid catabolic process), contractile regulation (cellular response to cAMP, chloride transport, inorganic ion transmembrane transport, cellular response to external stimulus, and action potential) and immune response (chemotaxis).

During the HFrEF stage, augmented immune response was one of the predominant biological processes comprising lymphocyte differentiation, regulation of inflammatory responses, interleukin-13 production, regulation of TNF superfamily cytokine production, positive regulation of immune response, regulation of cytokine production. Other enhanced processes included cardiac remodeling (multicellular organismal homeostasis, positive regulation of cell motility, and tissue morphogenesis) and lipid catabolic process (Figure 8E). In contrast, the suppressed biological processes primarily involved mitochondrial function (oxidative phosphorylation, NADH dehydrogenase complex assembly, proton transmembrane transport, mitochondrial electron transport-cytochrome c to oxygen, purine ribonucleotide biosynthetic process, mitochondrial electron transport NADH to ubiquinone, and mitochondrial electron transport ubiquinol to cytochrome c; Figure 8F). Other attenuated processes included cardiomyocyte growth (striated muscle cell differentiation) and lipid storage (monocarboxylic acid metabolic process and positive regulation of lipid storage; Figure 8F).

**FIGURE 9:**
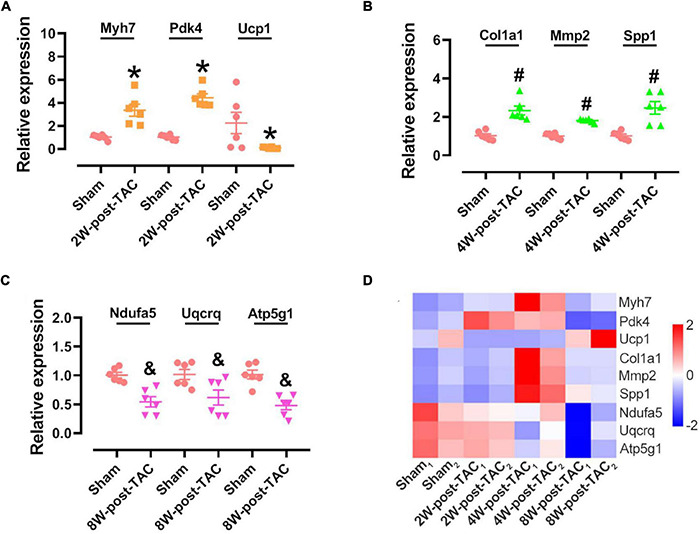
Hub gene identification in the 2W-, 4W-, and 8W-post TAC hearts. Nine genes were randomly selected for verifying their expression in the 2W-post-TAC **(A)**, 4W-post-TAC **(B)**, or 8W-post-TAC stage **(C)**. *N* = 6. *, ^#^, and ^&^ *P* < 0.05 vs. Sham. RNA sequencing profiles showed expression of these genes were dynamic in the three stages of heart failure **(D)**. *N* = 2 for the sham, 2W-, 4W-, and 8W-post-TAC groups. The color bar from blue to red, indicating expression levels from low to high.

Nine genes from three stages (three in each stage) were randomly selected to verify their expression using qPCR. We found that the results from GSEA and qPCR verification were consistent. The expression of *Myh7* and *Pdk4* was upregulated and that of *Ucp1* was downregulated in the hypertrophic stage (Figure 9A); the expression of *Col1a1*, *Mmp2*, and *Spp1* was upregulated (Figure 9B) in the HFpEF stage, and the expression of *Ndufa5*, *Uqcrq*, and *Atp5g1* was downregulated in the HFrEF stage (Figure 9C). The expression of these nine genes was dynamic in the three stage of heart failure (Figure 9D).

Furthermore, the top 40 hub genes were identified by PPI network analysis in the three stages of heart failure (Figure 5C). Pathway enrichment analysis revealed that downregulated genes were enriched in the PPAR signaling pathway, regulation of lipolysis in adipocytes, fatty acid metabolism, AMPK signaling pathway, and relaxin singling pathway in the hypertrophic stage. Cardiac remodeling became predominate in the HFpEF stage as evidenced by the augmented expression of genes enriched in protein digestion and absorption, ECM-receptor interaction, and TGF-beta pathways. When the pressure overload lasted for 8 weeks, the downregulation of oxidative phosphorylation and cardiac contraction was predicted (Table 2).

**TABLE 2:** KEGG pathway enrichment of top 40 hub genes.

KEGG pathways	Up/down	Genes	Log (*P*-value)	Log (*q*-value)
**2W-post-TAC vs. Sham**				
PPAR signaling pathway	Down	Adipoq, Pck1, Scd1 (*), Ucp1, Slc27a2, Plin1, Acaa1b	–9.775	–7.245
Regulation of lipolysis in adipocytes	Down	Adrb3 (*), Tshr, Plin1, Nppa, Adcy1 (*)	–7.357	–5.178
Fatty acid metabolism	Down	Fasn, Scd1 (*), Acaca (*), Elovl6 (*), Acaa1b	–7.132	–5.178
AMPK signaling pathway	Down	Adipoq, Fasn, Lep (*), Pck1, Scd1 (*), Acaca (*)	–7.106	–5.178
Relaxin signaling pathway	Down	Gng2 (*), Mmp9 (*), Rxfp1, Adcy1 (*)		
**4W-post-TAC vs. Sham**				
Protein digestion and absorption	Up	Col11a1, Col12a1, Col14a1, Col15a1, Col18a1, Col3a1, Col4a1, Col4a2, Col4a3 (*), Col5a1 (*), Col5a2, Col6a1 (*), Col6a2 (*), Col8a1, Col1a1 (*), Col1a2 (*), Eln, Col16a1, Col28a1, Col8a2, Col27a1 (*)	–38.016	–35.486
ECM-receptor interaction	Up	Col4a1, Col4a2, Col4a3 (*), Col6a1 (*), Col6a2 (*), Col1a1 (*), Col1a2 (*), Fn1, Spp1, Col3a1, Mmp2 (*), Tgfb2, Mmp9	–13.223	–10.993
TGF-beta signaling	Up	Fbn1, Fmod, Tgfb2	–3.221	–1.696
**8W-post-TAC vs. Sham**				
Oxidative phosphorylation	Down	Atp5g1, Atp5j, Cox6a2, Cox6c, Cox7a1, Cox7a2, Cycs, ATP6, Cox1, Cox2, Cox3, Cytb, Nd1, Nd2, Nd3, Nd4, Ndufa2, Uqcrq (*), Atp5j2, Ndufa3, Cox7b, Ndufc1, Ndufa12, Uqcr11, Ndufa5, Ndufa8, Ndufv3, Cox6b1, Ndufs6, Ndufs5	–63.267	–60.738
Cardiac muscle contraction	Down	Cox6a2, Cox6c, Cox7a1, Cox7a2, Cox1, Cox2, Cox3, Cytb, Uqcrq (*), Cox7b, Uqcr11, Cox6b1	–20.311	–18.860
Cytokine-cytokine receptor interaction	Up	Ccr6, Cxcl10, Il33 (*)	–4.022	–1.492

N = 2 for each of the sham and 2W-, 4W-, and 8W-TAC groups. Up, upregulated; Down, downregulated. * Predicted target genes of circRNAs.

In summary, mRNA expression identification demonstrated that cardiac remodeling and dysregulation of immune response and metabolism were the key events in all three stages of heart failure. Enrichment analysis of hub genes revealed that metabolism downregulation was the central event in the hypertrophic stage, cardiac remodeling was predominant in the HFpEF stage, and impairment in oxidative phosphorylation and cardiac muscle contraction was predicted in the HFrEF stage.

### Differential Circular RNA-Based Competitive Endogenous RNA Networks in All Three Stages of Pressure Overload-Induced Heart Failure

**FIGURE 10:**
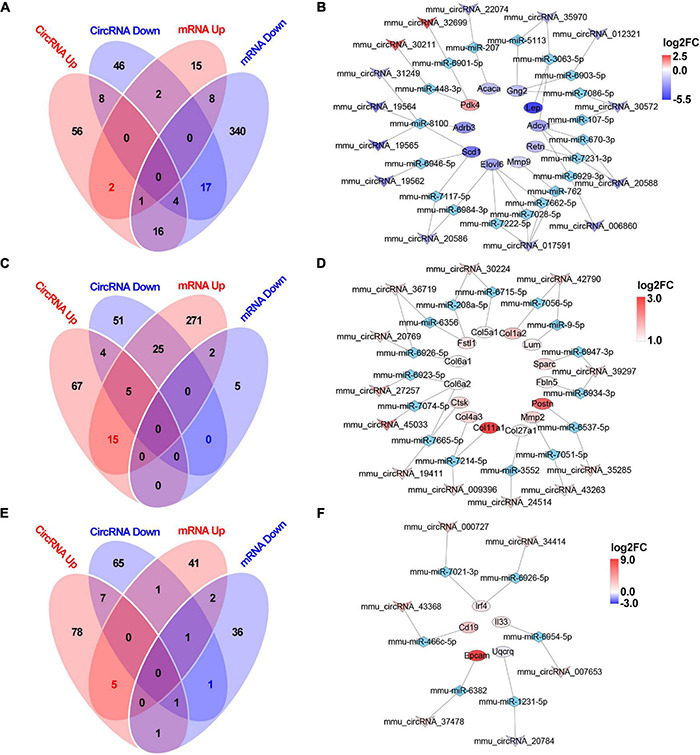
Circular RNA-based ceRNA networks in the 2W-, 4W- and 8W-post TAC hearts. The miRNAs interacting with the mRNAs or circRNAs in the 2W-post-TAC hypertrophic stage were displayed in panel **(A)**. The constructed ceRNA networks illustrated that the target mRNAs encoding adrenergic signaling, pyruvate metabolism, metabolic regulators, and matrix metallopeptidase in the hypertrophic hearts **(B)**. The miRNAs in the 4W-post-TAC HFpEF stage were displayed in panel **(C)**. The circRNAs were predicted to regulate mRNAs associated with collagens and matrix regulators, and adipokine **(D)**. The miRNAs in the 8W-post-TAC HFrEF stage were displayed in panel **(E)**. The circRNAs were predicted to regulate mRNAs associated with inflammatory regulation and a mitochondrial enzyme **(F)**. *N* = 2 for the sham, 2W-, 4W-, and 8W-post-TAC groups. Red, upregulated mRNAs modulated by circRNA-based ceRNA network; blue, downregulated mRNA modulated by circRNA-based ceRNA network; the colors from deep to light reflect the *P*-value from high to low.

After analyzing the interactions between the hub gene miRNAs and mRNAs and predicting the interactions between the circRNAs and miRNAs (Figure 5C), the miRNAs that interact with the mRNAs or circRNAs were determined. For the 2W-post-TAC hypertrophic stage, 28 and 386 miRNAs were predicted in the “mRNA up” and “mRNA down” groups, respectively, and 87 and 77 miRNAs in the “circRNA up” and “circRNA down” groups, respectively (Figure 10A). Subsequent ceRNA network analyses predicted the functions of circRNAs in mRNA regulation (Figure 10B). For the 2W-post-TAC hypertrophic stage, the circRNAs targeted the biological processes associated with adrenergic signaling (adrenergic receptors, G-protein coupling receptor, and adenylate cyclase; Figure 10B). These circRNAs and their target mRNAs (encoding proteins) were mmu_circRNA_006860 (*Adcy1*; adenylate cyclase 1), mmu_circRNA_012321 (*Gng2*; G protein gamma 2), mmu_circRNA_19564 (*Adrb3*; adrenergic receptor beta 3), mmu_circRNA_19565 (*Adrb3*), mmu_circRNA_20588 (*Adcy1*), mmu_circRNA_20588 (*Adcy1*), mmu_circRNA_30572 (*Adcy1*), mmu_circRNA_30572 (*Gng2*), mmu_circRNA_31249 (*Adrb3*), mmu_circRNA_35970 *(Adcy1*), and mmu_circRNA_35970 (*Gng2*). The mRNAs encoding proteins related to metabolism or metabolic regulation were targeted by mmu_circRNA_017591 (*Elovl6*; elongation of long chain fatty acids family member 6), mmu_circRNA_017591 (*Elovl6*), mmu_circRNA_017591 (*Elovl6*), mmu_circRNA_19562 (*Scd1*; stearoyl-Coenzyme A desaturase 1), mmu_circRNA_19564 (*Scd1*), mmu_circRNA_19565 (*Scd1*), mmu_circRNA_20586 (*Elovl6*), mmu_circRNA_20586 (*Scd1;* stearoyl-Coenzyme A desaturase 1), mmu_circRNA_22074 (*Acaca;* acetyl-Coenzyme A carboxylase alpha), mmu_circRNA_30211 (*Pdk4*; pyruvate dehydrogenase), mmu_circRNA_31249 (*Scd1*), and mmu_circRNA_32699 (*Pdk4*). In addition, mmu_circRNA_017591 targeted *Mmp9*, encoding protein matrix metallopeptidase 9. Adipokines (*Retn;* resistin) and (*Lep;* leptin) were targeted by mmu_circRNA_20588 and mmu_circRNA_35970, respectively. The chromosomal location, classification, and corresponding linear transcripts of the circRNAs in the hypertrophic hearts are listed in Table 3.

**TABLE 3:** Top 40 significantly dysregulated circular RNAs (circRNAs) in 2W-post-TAC mouse heart.

circRNA	Type	FC	Location	Corresponding linear transcript (gene symbol)
**Upregulated**				
mmu_circRNA_26225	a	6.726	chr13:42709703–42709850	NM_001302635 (Phactr1)
mmu_circRNA_41535	a	6.435	chr7:61813261–61913335	TCONS_0002942 (XLOC_022800)
mmu_circRNA_37478	a	6.274	chr4:116611971–116622814	NM_016777 (Nasp)
mmu_circRNA_45033	a	5.607	chr9:106239531–106241785	NM_020559 (Alas1)
mmu_circRNA_20182	a	5.224	chr1:60006233–60014018	NM_027407 (Ica1l)
mmu_circRNA_20843	a	4.958	chr1:129758445–129822695	NM_172485 (Thsd7b)
mmu_circRNA_30114	a	4.948	chr16:94611418–94686603	NM_007890 (Dyrk1a)
mmu_circRNA_004768	b	4.659	chr19:8874187–8874255	NM_146093 (Ubxn1)
mmu_circRNA_007784	a	4.644	chr9:77957140–77961053	NM_134255 (Elovl5)
mmu_circRNA_41526	c	4.497	chr7:61276438–61282828	uc009hey.1 (DOKist4)
mmu_circRNA_39663	a	4.485	chr5:145211745–145218883	NM_016683 (Zkscan5)
mmu_circRNA_27257	a	4.458	chr14:26608089–26612061	NM_145969 (Dennd6a)
mmu_circRNA_000233	a	4.382	chr6:135922871–135923470	NM_008171 (Grin2b)
mmu_circRNA_32699	a	4.332	chr19:46371935–46372375	NM_029186 (Tmem180)
mmu_circRNA_28074	a	4.179	chr14:121973608–121994470	NM_026861 (Ubac2)
mmu_circRNA_24650	b	4.127	chr12:5561603–5607431	ENSMUST00000175951 (Gm24758)
mmu_circRNA_45034	a	4.051	chr9:106295140–106308033	NM_027354 (Poc1a)
mmu_circRNA_32628	a	4.018	chr19:42991109–43013218	NM_001081257 (Hpse2)
mmu_circRNA_30211	a	4.009	chr17:8898860–8953923	NM_011866 (Pde10a)
mmu_circRNA_25020	a	4.003	chr12:44387899–44456702	NM_176930 (Nrcam)
**Downregulated**				
mmu_circRNA_19565	b	15.337	chr1:4197533–4231144	TCONS_00000350 (XLOC_000763)
mmu_circRNA_19564	b	11.836	chr1:4197533–4228619	TCONS_00000350 (XLOC_000763)
mmu_circRNA_30573	a	10.218	chr17:49707509–49722344	NM_177052 (Kif6)
mmu_circRNA_19563	b	10.137	chr1:4163854–4231144	TCONS_00000349 (XLOC_000762)
mmu_circRNA_19562	b	8.620	chr1:4147811–4170404	TCONS_00000349 (XLOC_000762)
mmu_circRNA_20588	a	7.396	chr1:97923093–97944677	NM_013626 (Pam)
mmu_circRNA_006860	a	7.314	chr5:106635609–106636323	NM_026856 (Zfp644)
mmu_circRNA_42008	a	6.327	chr7:101342497–101344034	NM_019990 (Stard10)
mmu_circRNA_012434	b	6.315	chr9:16374948–16378254	NM_001080814 (Fat3)
mmu_circRNA_28239	a	5.990	chr15:11883297–11895804	NM_008728 (Npr3)
mmu_circRNA_30196	c	5.866	chr17:6137210–6139151	ENSMUST00000142030 (Tulp4)
mmu_circRNA_012321	a	5.739	chr8:67713682–67720953	NM_027626 (Psd3)
mmu_circRNA_30572	a	5.562	chr17:49686390–49839207	NM_177052 (Kif6)
mmu_circRNA_008009	c	5.294	chr17:6137210–6139156	ENSMUST00000142030 (Tulp4)
mmu_circRNA_017591	a	5.256	chr12:81104606–81106011	NM_022316 (Smoc1)
mmu_circRNA_19175	c	5.100	chr17:6137208–6139156	ENSMUST00000142030 (Tulp4)
mmu_circRNA_31249	c	5.078	chr18:9386734–9412753	uc012azd.1 (Ccny)
mmu_circRNA_22074	a	4.946	chr10:59451205–59456809	NM_001033259 (Mcu)
mmu_circRNA_20586	a	4.889	chr1:97894360–97898389	NM_013626 (Pam)
mmu_circRNA_35970	a	4.873	chr3:108324595–108340705	NM_019972 (Sort1)

*Type a, exonic; type b, sense overlapping; type c, intronic. N = 2.*

For the 4W-post-TAC HFpEF stage, 318 and 7 miRNAs were predicted in the “mRNA up” and “mRNA down” groups, respectively, and 91 and 85 miRNAs were predicted in the “circRNA up” and circRNA down” groups, respectively (Figure 10C). The ceRNA network analyses predicted that the circRNAs regulated mRNAs primarily associated with extracellular matrix activity (Figure 10D). These circRNAs and their target mRNAs were mmu_circRNA_009396 (*Col11a1*; collagen type I alpha 1), mmu_circRNA_009396 (*Col4a3*; collagen type IV alpha 3), mmu_circRNA_19411 (*Col6a2*; collagen type XI alpha 1), mmu_circRNA_19411 (*Ctsk*), mmu_circRNA_20769 (*Col6a1*; collagen type XI alpha 1), mmu_circRNA_24514 (*Col27a1*; collagen type XXVII alpha 1), mmu_circRNA_27257 (*Col6a2*), mmu_circRNA_30224 (*Col5a1;* collagen type V alpha 1), mmu_circRNA_35285 (*Postn*; periostin), mmu_circRNA_36719 (*Col6a1*), mmu_circRNA_39297 (*Fbln5;* fibulin 5), mmu_circRNA_39297 (*Sparc;* secreted acidic cysteine rich glycoprotein), mmu_circRNA_42790 (*Col1a2;* collagen type I alpha 2), mmu_circRNA_42790 (*Lum;* lumican), circRNA_43263 (*Mmp2;* matrix metallopeptidase 2), and mmu_circRNA_45033 (*Col6a2*). In addition, circRNA_30224 and mmu_circRNA_36719 targeted *Fstl1*, encoding protein cytokine follistatin-like 1. The chromosomal location, classification, and corresponding linear transcripts of the circRNAs in the HFpEF hearts are listed in Table 4.

**TABLE 4:** Top 40 significantly dysregulated circRNAs in 4W-post-TAC mouse heart.

circRNA	Type	FC	Location	Corresponding linear transcript (gene symbol)
**Upregulated**				
mmu_circRNA_45033	a	4.444	chr9:106239531–106241785	NM_020559 (Alas1)
mmu_circRNA_27257	a	3.190	chr14:26608089–26612061	NM_145969 (Dennd6a)
mmu_circRNA_30751	a	2.945	chr17:64287000–64297909	NM_001025309 (Pja2)
mmu_circRNA_42790	a	2.875	chr8:35806996–35812434	TCONS_00032238 (XLOC_023637)
mmu_circRNA_37457	a	2.860	chr4:115752920–115766512	NM_172698 (Efcab14)
mmu_circRNA_42789	a	2.831	chr8:35806707–35812434	TCONS_00032237 (XLOC_023637)
mmu_circRNA_37478	a	2.831	chr4:116611971–116622814	NM_016777 (Nasp)
mmu_circRNA_30224	a	2.816	chr17:10273946–10282981	NM_021881 (Qk)
mmu_circRNA_39297	a	2.815	chr5:121879814–121887488	NM_007804 (Cux2)
mmu_circRNA_35285	a	2.767	chr3:40672570–40675344	NM_175515 (Intu)
mmu_circRNA_24514	a	2.759	chr11:121623431–121630867	NM_178664 (B3gntl1)
mmu_circRNA_23283	a	2.740	chr11:50071170–50087461	NM_021540 (Rnf130)
mmu_circRNA_19411	b	2.738	chr6:31418929–31433132	NM_013791 (Mkln1)
mmu_circRNA_20769	b	2.663	chr1:125572314–125572446	NM_028787 (Slc35f5)
mmu_circRNA_009396	a	2.653	chr6:31418930–31433131	NM_013791 (Mkln1)
mmu_circRNA_37422	a	2.636	chr4:109361669–109380029	NM_007943 (Eps15)
mmu_circRNA_36719	a	2.612	chr4:45444678–45447677	NM_001033306 (Shb)
mmu_circRNA_22066	a	2.488	chr10:58983920–59007137	NM_172788 (Sh3rf3)
mmu_circRNA_007990	a	2.471	chr7:112759383–112759639	NM_009346 (Tead1)
mmu_circRNA_43263	b	2.427	chr8:86724205–86725856	NM_009172 (Siah1a)
**Downregulated**				
mmu_circRNA_19565	b	13.375	chr1:4197533–4231144	TCONS_00000350 (XLOC_000763)
mmu_circRNA_30573	a	13.021	chr17:49707509–49722344	NM_177052 (Kif6)
mmu_circRNA_19564	b	11.239	chr1:4197533–4228619	TCONS_00000350 (XLOC_000763)
mmu_circRNA_19563	b	9.997	chr1:4163854–4231144	TCONS_00000349 (XLOC_000762)
mmu_circRNA_42008	a	8.235	chr7:101342497–101344034	NM_019990 (Stard10)
mmu_circRNA_012321	a	7.561	chr8:67713682–67720953	NM_027626 (Psd3)
mmu_circRNA_19562	b	7.453	chr1:4147811–4170404	TCONS_00000349 (XLOC_000762)
mmu_circRNA_30572	a	6.394	chr17:49686390–49839207	NM_177052 (Kif6)
mmu_circRNA_012434	b	5.158	chr9:16374948–16378254	NM_001080814 (Fat3)
mmu_circRNA_011031	a	4.986	chr3:120772681–120799747	TCONS_00021080 (XLOC_016164)
mmu_circRNA_23203	a	4.966	chr11:35588932–35599487	NM_011412 (Slit3)
mmu_circRNA_35970	a	4.815	chr3:108324595–108340705	NM_019972 (Sort1)
mmu_circRNA_23205	a	4.582	chr11:35622007–35634034	NM_011412 (Slit3)
mmu_circRNA_006013	a	4.436	chrX:69544408–69545269	NM_008032 (Aff2)
mmu_circRNA_45496	a	4.271	chrX:69523435–69545269	NM_008032 (Aff2)
mmu_circRNA_28239	a	4.140	chr15:11883297–11895804	NM_008728 (Npr3)
mmu_circRNA_29154	a	4.112	chr16:5097644–5102678	NM_008909 (Ppl)
mmu_circRNA_017690	a	3.996	chr4:13835632–13846957	NM_009822 (Runx1t1)
mmu_circRNA_40134	a	3.925	chr6:37353556–37358647	NM_178661 (Creb3l2)
mmu_circRNA_31193	c	3.689	chr18:5210876–5214919	AK020518 (–)

*Type a, exonic; type b, sense overlapping; type c, intronic. N = 2.*

For the 8W-post-TAC HFrEF stage, 50 and 42 miRNAs were predicted in the “mRNA up” and “mRNA down” groups, respectively, and 92 and 76 miRNAs in the “circRNA up” and “circRNA down” groups, respectively (Figure 10E). The ceRNA network analyses predicted that, in the 8W-post-TAC EF-reduced hearts, the circRNAs would regulate mRNAs primarily associated with immune response (Figure 10F). These circRNAs and their target mRNAs (encoding proteins) were mmu_circRNA_000727 (*Irf4*; interferon regulatory factor 4), mmu_circRNA_007653 *(Il33;* interleukin 33), mmu_circRNA_34414 (*Irf4*), mmu_circRNA_37478 (*Epcam;* epithelial cell adhesion molecule), and mmu_circRNA_43368 (*Cd19*; CD19 antigen). In addition, a circRNA, mmu_circRNA_20784 was found to target *Uqcrq*, encoding mitochondrial protein ubiquinol-cytochrome c reductase complex III subunit VII. The chromosomal location, classification, and corresponding linear transcripts of the circRNAs in the HFrEF hearts are listed in Table 5.

**TABLE 5:** Top 40 significantly dysregulated circRNAs in 8W-post-TAC mouse heart.

circRNA	Type	FC	Location	Corresponding linear transcript (gene symbol)
**Upregulated**				
mmu_circRNA_43368	d	10.114	chr8:96354394–96358728	–
mmu_circRNA_34947	a	8.938	chr2:164799719–164799945	NM_133240 (Acot8)
mmu_circRNA_43449	b	8.610	chr8:109853672–109861329	ENSMUST00000173980 (Gm17344)
mmu_circRNA_34800	a	8.409	chr2:154345865–154348781	NM_025876 (Cdk5rap1)
mmu_circRNA_35219	a	8.119	chr3:32437117–32442831	NM_008839 (Pik3ca)
mmu_circRNA_31781	a	7.803	chr18:57897343–57915508	NM_009194 (Slc12a2)
mmu_circRNA_24822	a	7.668	chr12:28524674–28535580	NM_001177964 (Dcdc2c)
mmu_circRNA_000727	a	7.654	chr1:153482741–153483872	NM_007842 (Dhx9)
mmu_circRNA_19176	b	7.429	chr17:14986716–15048657	NM_001039552 (Ermard)
mmu_circRNA_43882	b	6.027	chr9:23406494–23422050	NM_028472 (Bmper)
mmu_circRNA_35338	a	6.013	chr3:51247804–51248074	NM_009834 (Ccrn4l)
mmu_circRNA_40761	a	5.719	chr6:113289984–113295153	NM_170673 (Cpne9)
mmu_circRNA_36867	b	5.653	chr4:59420842–59424150	NM_001163288 (Susd1)
mmu_circRNA_42066	a	5.306	chr7:109824764–109833125	NM_020052 (Scube2)
mmu_circRNA_28128	a	4.568	chr14:123827751–123857579	NM_145467 (Itgbl1)
mmu_circRNA_007653	a	4.495	chr9:20891082–20891206	NM_145610 (Ppan)
mmu_circRNA_34414	a	4.351	chr2:121298596–121299046	NM_032393 (Map1a)
mmu_circRNA_37478	a	4.182	chr4:116611971–116622814	NM_016777 (Nasp)
mmu_circRNA_38377	a	4.028	chr5:31169835–31170274	NM_027901 (Gtf3c2)
mmu_circRNA_42044	a	4.019	chr7:105564971–105567753	NM_009685 (Apbb1)
**Downregulated**				
mmu_circRNA_22074	a	6.702	chr10:59451205–59456809	NM_001033259 (Mcu)
mmu_circRNA_008009	c	5.691	chr17:6137210–6139156	ENSMUST00000142030 (Tulp4)
mmu_circRNA_19175	c	5.450	chr17:6137208–6139156	ENSMUST00000142030 (Tulp4)
mmu_circRNA_30572	a	4.366	chr17:49686390–49839207	NM_177052 (Kif6)
mmu_circRNA_011865	a	4.131	chr5:106634830–106666845	NM_026856 (Zfp644)
mmu_circRNA_20784	a	4.098	chr1:127397506–127412260	NM_145128 (Mgat5)
mmu_circRNA_42820	a	4.011	chr8:41048047–41084840	NM_001005863 (Mtus1)
mmu_circRNA_000476	a	3.987	chr13:55233894–55248077	NM_008739 (Nsd1)
mmu_circRNA_19094	b	3.939	chr13:55233892–55248077	NM_008739 (Nsd1)
mmu_circRNA_38983	a	3.910	chr5:92460041–92462084	NM_007644 (Scarb2)
mmu_circRNA_27197	a	3.820	chr14:21774962–21780181	NM_026283 (Samd8)
mmu_circRNA_32197	b	3.792	chr19:5797448–5798900	NR_002847 (Malat1)
mmu_circRNA_39099	c	3.541	chr5:106634830–106669840	ENSMUST00000125895 (Zfp644)
mmu_circRNA_39100	c	3.481	chr5:106634830–106679743	ENSMUST00000125895 (Zfp644)
mmu_circRNA_014393	a	3.377	chr5:106634830–106638635	NM_026856 (Zfp644)
mmu_circRNA_19242	b	3.298	chr2:37640848–37647288	NM_009261 (Strbp)
mmu_circRNA_43679	a	3.229	chr9:3441054–3460131	NM_027545 (Cwf19l2)
mmu_circRNA_38982	a	3.160	chr5:92454039–92454851	NM_007644 (Scarb2)
mmu_circRNA_009340	b	3.141	chr13:23739295–23739355	NM_015786 (Hist1h1c)
mmu_circRNA_004058	a	3.084	chr4:84971130–85006524	NM_175275 (Cntln)

*Type a, exonic; type b, sense overlapping; type c, intronic; type d, intergenic. N = 2.*

Overall, there are distinct circRNA-based ceRNA networks in hypertrophy, HFpEF, and HFrEF. circRNAs were predicted to regulate cardiac metabolism, remodeling, and mitochondrial function/cardiac contraction in hypertrophy, HFpEF, and HFrEF, respectively, induced by pressure overload.

## Discussion

**FIGURE 11:**
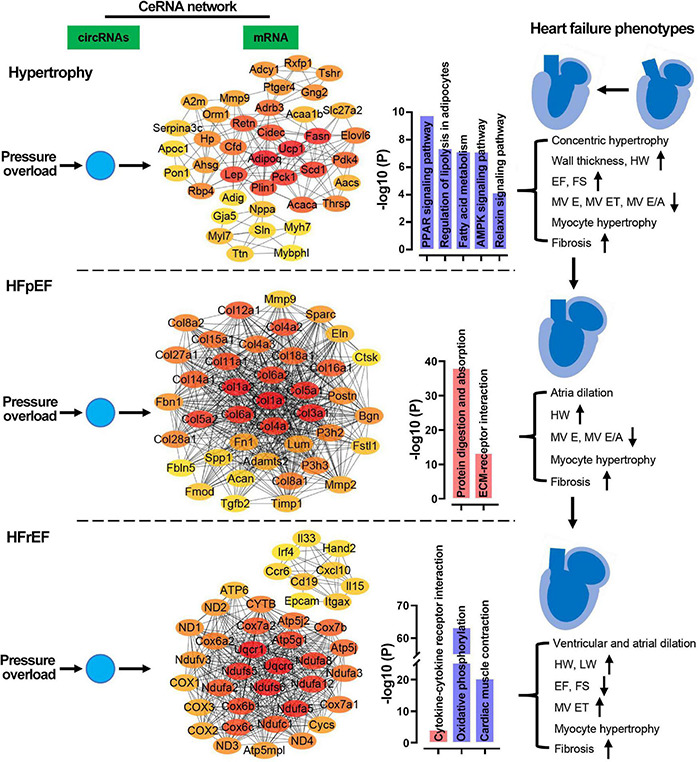
Potential regulatory mechanisms of ceRNA networks in the three stages of heart failure. In the pressure overload hearts, circRNAs were predicted to regulate cardiac metabolism, remodeling, and mitochondrial function/cardiac contraction in hypertrophy, HFpEF, and HFrEF, respectively. HW, heart weight; LW, lung weight; EF, ejection fraction of the left ventricle; FS, fractional shortening; MV ET, ejection time of the left ventricle; IVCT, isovolumetric contraction time; IVRT, isovolumetric relaxations time; MV E/A, the ratio of peak E-wave to peak A-wave velocity from mitral inflow Doppler.

In the present study, we characterized the morphological, functional, and pathological changes occurring in murine heart failure progression and identified distinct hypertrophic, HFpEF, and HFrEF stages in pressure overload hearts. After pressure overload, heart weight, cardiomyocyte hypertrophy, and fibrosis gradually increased over time. Typically, in the early hypertrophic stage, hearts displayed concentric hypertrophy, thickened left ventricular walls, increased EF and FS, and reduced MV E and MV ET. The HFpEF stage was characterized by preserved EF and FS, dilated atria, increased LV systolic volume, and reduced MV E. The advanced HFrEF stage typically presented with ventricular and atrial dilation, increased LV systolic and diastolic volume, reduced EF and FS, and increased MV ET. In the biological process enrichment analysis of mRNAs, we found that pressure overload induced cardiac remodeling, immune response dysregulation, and metabolic disorder. Contractile regulation of the heart was interfered with in the hypertrophic and HFpEF stages. The long-term pressure overload led to mitochondrial dysfunction. The ceRNA network showed that circRNAs predominately targeted metabolism in the hypertrophic stage, while targeting predominately cardiac remodeling in the HFpEF stage, and mitochondrial oxidation and cardiac muscle contraction in the HFrEF stage. Our results showed that circRNA-based ceRNA networks were crucial to heart failure phenotypes induced by pressure overload. The distinct regulatory roles of the circRNA-based ceRNA network in the three heart failure stages are summarized in Figure 11.

### Identification of the Key Features of the Heart Failure Stages Facilitates the Precise Application of the Transverse Aortic Constriction Model

Here, we applied the conventional open-chest technique to induce pressure overload. Though it has been slightly modified in recent years (Zaw et al., 2017; Eichhorn et al., 2018), the open chest procedure remains the most frequently applied method for inducing chronic pressure overload. In addition, the development of high-resolution echocardiography has enabled the precise evaluation of heart structure and function in biomedical research conducted on small animals, such as rodents. We found that murine hearts recapitulated hypertrophy, HFpEF, and HFrEF after 2, 4, and 8 weeks of TAC stress. The diverse remodeling phenotypes observed in our murine TAC model resembled those seen in human hypertensive patients. For example, enlargement of the left atria was detected in the 4- and 8-week post-TAC groups; the prevalence of which is between 16 and 83% among hypertensive patients (Cuspidi et al., 2013) and is associated with chronic pressure overload and diastolic dysfunction (Douglas, 2003; Leung et al., 2008).

Hypertensive myopathy is classified into four categories based on its progression. In Degree I, isolated LV diastolic dysfunction occurs with no LV hypertrophy. In Degree II, LV diastolic dysfunction occurs with concentric hypertrophy. In Degree III, clinical heart failure is observed (dyspnea, pulmonary edema, and preserved ejection fraction). In Degree IV, dilated cardiomyopathy with heart failure and reduced ejection fraction occurs (Iriarte et al., 1993; Messerli et al., 2017). Accordingly, we divided the TAC model into three stages and found that the hypertrophic remodeling stage corresponds to Degree II, the HFpEF stage corresponds to Degree III, and the HFrEF stage corresponds to Degree IV. Hence, the TAC-induced pressure overload model is feasible and suitable for studying the different stages of heart failure.

Furthermore, based on the foregoing morphological, functional, and pathological changes, we propose that the 4W-post-TAC mice can serve as a model for HFpEF. The United States, Japan, and various European nations have recently adopted similar guidelines for heart failure diagnosis and treatment. They classified heart failure as reduced ejection fraction (HFpEF; LVEF ≤ 40%), mid-range EF (HFmrEF; LVEF = 40–49%), or preserved EF (HFpEF; LVEF ≥ 50%) (Yancy et al., 2013; Ponikowski et al., 2016; Tsutsui et al., 2019). In clinical practice, heart failure associated with mid-range LVEF is also commonly grouped into HFpEF. Approximately 50% of heart failure patients are categorized as HFpEF. Unlike HFrEF, the disease mechanisms associated with HFpEF are poorly understood and few animal models are available to identify its drug targets. However, we recently reported that aortic constriction induces HFpEF in pigs (Tan et al., 2021). Meanwhile, in the current study, we demonstrated that the classic murine TAC model presents the HFpEF stage during heart failure progression. Besides the preserved-EF, the failing hearts at this stage displayed atrial enlargement, cardiomyocyte hypertrophy, fibrosis, and gene expression profile shifts to immune response dysregulation and metabolic disorder.

### mRNA Expression Profiles Suggest Therapeutic Targets in the Three Stages of Heart Failure

Hypertensive heart failure results from interactions among mechanical loading and modifications of the cardiomyocyte phenotypes, the extracellular matrix, and intracellular signaling regulation. The major features of pressure overload-induced remodeling include myocyte hypertrophy, interstitial fibrosis, and collagen degradation (Messerli et al., 2017; González et al., 2018). Interstitial collagen fibers progressively accumulate in the hypertrophic ventricles of hypertensive patients (Rossi, 1998). Herein, interstitial fibrosis and myocyte hypertrophy first appeared in the hypertrophic stage and persisted through the HFpEF and HFrEF stages. The degree of cardiac fibrosis and cardiomyocyte size are routinely used to assess the effects of anti-hypertensive interventions in experimental models. For example, in mice with TAC-induced heart failure, berberine significantly reduces the cross-sectional area of hypertrophic cardiomyocytes induced by pressure overload (Abudureyimu et al., 2020). We found that the increases in cross-sectional cardiomyocyte area were dramatic whereas collagen volume only slowly expanded during heart failure progression. Thus, even small reductions in interstitial fibrosis during the early stages of heart failure can yield significant outcomes.

The immune response regulated by cytokines and chemokines is another key feature of heart failure in pressure overload hearts. NF-κB signaling ablation suppresses ventricular hypertrophy in TAC mice (Liu et al., 2012). Compared with the hypertrophic stage, inflammatory signaling pathways are more heavily implicated in the HFrEF stage. A gene enrichment further analysis revealed that six of the top ten upregulated biological processes were classified as immune responses. Upregulated transcripts associated with lymphocyte differentiation, regulation of inflammatory response, regulation of TNF superfamily cytokine, positive regulation of immune response, and regulation of cytokine production were identified (Figure 8E). Therefore, management of the inflammatory state is vital to the successful treatment of advanced heart failure induced by pressure overload. In addition, we found the HFrEF stage was accompanied by mitochondrial dysfunction. Evidence revealed that reduced mitochondrial respiration capacity was associated with heart failure, suggesting that mitochondrial abnormalities may represent therapeutic targets (Brown et al., 2017; Li et al., 2022). Furthermore, nicotinamide adenine dinucleotide (NAD^+^) supply inhibits inflammatory activation in heart failure (Zhou et al., 2020). Meanwhile, damaged mitochondria might be associated with inflammation *via* TLR signaling (Manfredi and Rovere-Querini, 2010). However, the interactions between mitochondrial damage and inflammation merit further investigation even though mRNA sequencing analysis has already profiled the pathways involved.

Mitochondria generate ATP primarily *via* the tricarboxylic acid (TCA) cycle and oxidative phosphorylation. Mitochondrial function is linked to cardiac contraction, cell growth, cell death, and inflammation (Kolwicz et al., 2013). In the present study, oxidative phosphorylation was depressed, and several key mitochondrial components associated with ATP generation [complex I (NADH dehydrogenase complex assembly), proton transport, electron transport (NADH to ubiquinone, ubiquinone to cytochrome c, cytochrome c to oxygen), and transporter (monocarboxylic acid metabolism)] were downregulated in remodeled hearts with functional failure. These findings were consistent with those of an earlier study wherein heart failure suppressed mitochondrial electron transport chain (ETC) complex function (Sung et al., 2010). Unlike the HFrEF stage, abnormal fatty acid and glucose metabolism predominate in the hypertrophy and HFpEF stages. Several approaches targeting mitochondria have been explored in heart failure treatment including interference with mitochondrial biogenesis, and antioxidant treatment, among others (Bayeva et al., 2013). Hence, mitochondrial function is a potential therapeutic target for heart failure characterized by dilated LV structure and reduced EF.

The murine TAC-induced hypertrophy model has been widely used for investigating molecular mechanisms and drug targets. An investigation into transient phenotypes in different time points or with different constriction degree showed that Myh7 expression was increased in myocardia with hypertrophy and HFpEF (van den Bosch et al., 2006; Melleby et al., 2018; Richards et al., 2019). Our results from gene profile analysis and expression verification for 2W-post-TAC myocardium were consistent with these previous findings. In addition, we found that Myh7 did not alter in the dilated HFrEF stage. We found that the expression of Col1a1 and Mmp2 increased in the HFpEF stage but not in the HFrEF stage, which is consistent with the findings showing that the protein expression of MMP2 was upregulated in the EF-preserved stage but returned to the sham level (Givvimani et al., 2010). However, some of our findings contradicted those of previous studies. For example, we found that the expression of Col1a1 was downregulated in dilatated hearts, while another group reported a upregulation result (Xiang et al., 2017). These results suggest that the regulation of gene expression is complicated and that investigating the dynamic changes in gene expression is crucial for understanding the progression of heart failure.

### Circular RNAs Regulate Cellular Function *via* the Competitive Endogenous RNA Network During Heart Failure Progression

We found that circRNAs potentially regulate adrenergic receptor signaling, lipid metabolism, extracellular matrix activity, and immune response during heart failure progression. To the best of our knowledge, this study is the first to profile circRNAs in the three stages of heart failure induced by pressure overload. Over the past two decades, researchers have implemented high-throughput microarray technology to characterize circRNA expression in physiological and pathological conditions. In the early 2010s, circRNAs were found to be widely expressed in mammal (Memczak et al., 2013). Researchers then identified circRNAs in human, mouse, and rat hearts (Werfel et al., 2016). Recent evidence suggests that circRNAs can be developed as biomarkers (Bei et al., 2018; Zhang et al., 2020). Moreover, several research groups have provided substantial evidence to demonstrate that circRNAs may exacerbate cardiac hypertrophy, induce cardiomyocyte senescence, regulate cellular matrix, and modulate cardiac repair in various cardiac pathological conditions. For example, circFoxo3 interacts with the inhibitor of DNA binding 1 (ILd1) and E2F transcription factor 1 (E2F1), thereby promoting cardiomyocyte senescence in doxorubicin-treated mice (Du et al., 2017). The same research group discovered that circYap inhibits actin polymerization in the cellular matrix (Wu et al., 2021). Three additional detrimental circRNAs were recently reported, namely, circRNA_000203, circNfix, and circNlgn. Downregulation of circNfix promotes angiogenesis and inhibits cardiomyocyte apoptosis (Huang et al., 2019). circRNA_000203 exacerbates cardiac hypertrophy by inhibiting miRNA expression (Li H. et al., 2020). Meanwhile, circNlgn was translated to neuroligin and found to promote remodeling in pressure overload hearts (Du et al., 2021). Furthermore, Tijsen et al. (2021) first reported that the contractile protein titin formed circRNAs (cTTN1) transcript is important for the splicing of several muscle genes including titin itself. Here, our findings have described a potentially new regulatory role for circRNAs. Garikipati et al. (2019) reported that a circular RNA mmu_circRNA_22074 (circMcu) is downregulated in post-MI hearts, while our ceRNA network analysis suggests that downregulation of this circular transcript is associated with the linear transcript Acaca encoding the alpha form of acetyl-CoA carboxylase in the hypertrophic stage. This enzyme catalyzes the carboxylation of acetyl-CoA in fatty acid biosynthesis (Brownsey et al., 2006). Hence, circRNAs play pivotal roles in regulating cellular metabolism in disease conditions and mediating their expression represents a potentially effective therapeutic strategy for metabolic heart disease. However, the regulation of circRNAs in heart failure progression remains largely unknown.

According to the CircAtlas, high-throughput and deep sequencing technologies have disclosed >413,000 and >175,000 circRNAs in humans and mice, respectively (Wu W. et al., 2020). While new circRNAs continue to be discovered,^5^ only a small proportion may, in fact, act intracellularly. Moreover, researchers may have focused on certain specific transcripts while discarding other potentially relevant sequencing or microarray data in disease mechanistic studies. Consequently, our understanding of the expression characteristics of these transcripts is impeded. Here, we detected ∼12,000 circRNAs in murine pressure overload hearts *via* an Illumina mouse circular RNA array, and these datasets have been deposited in the GEO database. Our analyses indicate that circRNAs potentially regulate heart failure progression by targeting immune response, cardiac remodeling, metabolism, mitochondrial function, and contractile regulation. Thus, further mechanistic studies are warranted to explore how these circRNAs regulate the cardiac remodeling and dysfunction, as well as to test whether these circRNAs can be developed as biomarkers or therapeutic targets.

### Limitations of the Study

In this study, we explored the roles of the ceRNA network in the regulation of circRNAs in cardiac remodeling and dysfunction in pressure overload hearts. However, besides the ceRNA regulatory mechanism, circRNAs can be translated into proteins or interact with RNA binding proteins. To fully understand their role in heart failure, the other functions of circRNAs need to be explored. Moreover, it remains unknown whether circRNAs have potential therapeutic efficacy, so further investigation into the regulatory mechanisms at the protein expression level is needed. Regarding the mouse sub-strain C57BL/6J used in the study, its phenotypes differs from that of sub-strain C57BL/6N in response to TAC stress (Garcia-Menendez et al., 2013). Consequently, the roles of ceRNA regulation in heart failure progression might be distinct between the murine sub-strains. In addition, the animals selected here were all males, which leaves the potential mechanisms of female mice uninvestigated. Sex differences in cardiac remodeling and heart failure have been widely reported (Lam et al., 2019; Wu J. et al., 2020). Thus, with reference to the results reported in the present manuscript, the mouse sub-strain and the sex should be specified.

## Data Availability Statement

The datasets presented in this study can be found in online repositories. The names of the repository/repositories and accession number(s) can be found below: https://www.ncbi.nlm.nih.gov/geo/ GSE182912; GSE182985.

## Ethics Statement

The animal study was reviewed and approved by the Institutional Animal Care and Use Committee (IACUC) of the Guangdong Laboratory Animals Monitoring Institute (No. IACUC2017015).

## Author Contributions

FY, XL, and WT designed and initiated the project. WT performed the animal surgery. XL, SZ, HC, LK, and JW were responsible for cardiac imaging evaluation, pathological analysis, or animal care. XL and FY performed bioinformatics analysis, results interpretation, and manuscript preparation. WP, CZ, PB, and YZ provided critical comments during experiment design and manuscript preparation. All authors read and approved the final manuscript.

## Conflict of Interest

The authors declare that the research was conducted in the absence of any commercial or financial relationships that could be construed as a potential conflict of interest.

## Publisher’s Note

All claims expressed in this article are solely those of the authors and do not necessarily represent those of their affiliated organizations, or those of the publisher, the editors and the reviewers. Any product that may be evaluated in this article, or claim that may be made by its manufacturer, is not guaranteed or endorsed by the publisher.

## Footnotes

1
https://www.ncbi.nlm.nih.gov/geo/

2
https://metascape.org

3
https://www.string-db.org/

4
http://www.cytoscape.org

5
http://circatlas.biols.ac.cn/
