# Supplementary material for: Differential mRNA Expression and Circular RNA-Based Competitive Endogenous RNA Networks in the Three Stages of Heart Failure in Transverse Aortic Constriction Mice
Source: Front Physiol. 2022 Mar 7;13:777284. doi: 10.3389/fphys.2022.777284 (PMC8940230; doi:10.3389/fphys.2022.777284)
Supplement: Supplementary file 1 [file Table_1.DOCX]

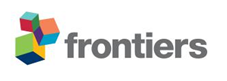


**Supplementary Table 1.** RT-qPCR Primers used in this study.

| **Gene** | **Forward** | **Reverse** |
| --- | --- | --- |
| **Myh7** | ATGAGACGGTGGTGGGTTTG | TTGCCTTTGCCTTTGTCCG |
| **Pdk4** | AAACCGTCCTTCCTTGACCC | TTGGAGCAGTGGAGTACGTG |
| **Ucp1** | CGGGCATTCAGAGGCAAATC | GAGGCAGGTGTTTCTCTCCC |
| **Col1a1** | GTGTTCCCTACTCAGCCGTC | ACTCGAACGGGAATCCATCG |
| **Mmp2** | AACGGTCGGGAATACAGCAG | TGGTAAACAAGGCTTCATGGG |
| **Spp1** | TTGCTTGGGTTTGCAGTCTTCTG | CTCATGGTCGTAGTTAGTCCCTC |
| **Ndufa5** | TCCACACGAGGAGCCAGATG | TCCACTGGTTAGCAGGCGG |
| **Uqcrq** | TCTCCTACAGCTTGTCGCCC | GCTCAAACTCCTGGTTGCC |
| **Atp5g1** | AGTGGGAGTGCAGATTGAAA | CAGGAAGGCTGCTTAGATGG |
